# Preparation and characteristics of gelatin sponges crosslinked by microbial transglutaminase

**DOI:** 10.7717/peerj.3665

**Published:** 2017-08-09

**Authors:** Haiyan Long, Kunlong Ma, Zhenghua Xiao, Xiaomei Ren, Gang Yang

**Affiliations:** 1 Center of Engineering-Training, Chengdu Aeronautic Polytechnic, Chengdu, China; 2 Department of Orthopaedics, Yongchuan Hospital, Chongqing Medical University, Chongqing, China; 3 Department of Cardiovascular Surgery, West China Hospital, Sichuan University, Chengdu, China; 4 Department of Medical Information and Engineering, School of Electrical Engineering and Information, Sichuan University, Chengdu, China

**Keywords:** Gelatin, Crosslink, Transglutaminase, Stem cells, Sponge

## Abstract

Microbial transglutaminase (mTG) was used as a crosslinking agent in the preparation of gelatin sponges. The physical properties of the materials were evaluated by measuring their material porosity, water absorption, and elastic modulus. The stability of the sponges were assessed via hydrolysis and enzymolysis. To study the material degradation *in vivo*, subcutaneous implantations of sponges were performed on rats for 1–3 months, and the implanted sponges were analyzed. To evaluate the cell compatibility of the mTG crosslinked gelatin sponges (mTG sponges), adipose-derived stromal stem cells were cultured and inoculated into the scaffold. Cell proliferation and viability were measured using alamarBlue assay and LIVE/DEAD fluorescence staining, respectively. Cell adhesion on the sponges was observed by scanning electron microscopy (SEM). Results show that mTG sponges have uniform pore size, high porosity and water absorption, and good mechanical properties. In subcutaneous implantation, the material was partially degraded in the first month and completely absorbed in the third month. Cell experiments showed evident cell proliferation and high viability. Results also showed that the cells grew vigorously and adhered tightly to the sponge. In conclusion, mTG sponge has good biocompatibility and can be used in tissue engineering and regenerative medicine.

## Introduction

Tissue engineering aims to repair and reconstruct damaged tissues and organs. Biological scaffolds designed for tissue engineering not only provide mechanical support for cell growth but also provide a micro environment that can regulate cell behavior and tissue regeneration. One of the challenges in scaffold design is in ensuring good mechanical structure and properties, rich active groups, as well as excellent biocompatibility and biodegradability. In addition, the design of biomaterials should mimic the physical characteristics and biological attributes of natural extracellular matrix (ECM) as much as possible (Bencherif et al., 2012; Bencherif, Braschler & Renaud, 2013; Kennedy et al., 2014; Bencherif et al., 2015; Xiao et al., 2015).

ECM is a secretory product of cells, which can form highly ordered insoluble aggregates that combine with cells to form various tissues and organs. ECM provides a 3D space for cell growth, affects cell adhesion, migration, proliferation, apoptosis, and signal transduction, and is involved in the development of organisms and the process of inflammatory response. Collagen is the major structural protein of ECM. *In vivo*, they can self-assemble to form collagen fibers of highly ordered three-helix structure with high mechanical strength.

Gelatin is a hydrolysis product of collagen, which retains many functional groups that can be identified by cells. Therefore, gelatin is good for cell adhesion. Gelatin also has good hydrophilicity, biodegradability, and low antigenicity. Thus, it is a popular source of many composite biomaterials. However, pure gelatin material is brittle and soluble in water, which hinders its application in tissue engineering. To date, findings of previous work show that the mechanical and thermal properties of gelatin material can be improved by crosslinking gelatin molecules. The crosslinking methods include physical crosslink (dehydrothermal treatment (Nadeem et al., 2013; Siimon et al., 2014); UV irradiation (Bhat & Karim, 2014); chemical crosslink, such as glutaraldehyde (Dainiak et al., 2010; Ito et al., 2014; Poursamar et al., 2016), carbodiimide (Pieper et al., 1999; Chimenti et al., 2011; Yeh et al., 2012), and genipin (Nadeem et al., 2013; Poursamar et al., 2016); enzymatical crosslink, such as transglutaminase (Yung et al., 2007; Kuwahara et al., 2010; Yamamoto et al., 2013) and horseradish peroxidase (Sakai et al., 2009; Chuang et al., 2015)). Physical crosslinking methods, such as dehydrothermal treatment, may cause degeneration of gelatin protein. Furthermore, UV irradiation does not easily penetrate thick material, leading to incomplete crosslinking. In the use of chemical crosslinking agents, the side effects of residual crosslinkers in scaffolds need to be considered. Chemical crosslinking agents, such as glutaraldehyde, glycerin aldehyde, formaldehyde, epoxy resin, and carbodiimide, have cytotoxicity, which affects cell growth and even lead to cell apoptosis.

Transglutaminase has a remarkable ability of protein crosslinking. This enzyme catalyzes acyl-transfer reactions between λ-carboxyamide groups of glutamine residues and ε-amino groups of lysine residues, resulting in the formation of ε-(λ-glutaminyl) lysine intra and intermolecular crosslinked proteins (Halloran et al., 2008). In the past, mammalian-derived transglutaminase was limited by its high production cost. At present, the transglutaminase extracted from microorganisms have high yield and low price, which make it suitable as a crosslinking agent (Collighan & Griffin, 2009). Our previous study (Yang et al., 2016) has shown that mTG crosslinked gelatin hydrogel has good biocompatibility and can support the growth and proliferation of adipose-derived stromal stem cells (ADSCs). We hypothesized that the preparation of mTG crosslinked gelatin sponges (mTG sponges) by freeze drying may also be suitable for tissue engineering applications. To the best of our knowledge, no study has been conducted from this perspective.

In the current study, mTG was used as a crosslinking agent in the preparation of porous gelatin sponges. The physical properties of the sponges were evaluated by measuring their material porosity, water absorption, and elastic modulus. The stability of the sponges were assessed via hydrolysis and enzymolysis. To study the material degradation *in vivo*, the morphology and histochemical staining of the subcutaneously implanted sponges were analyzed in the animal experiments of rats for 1–3 months. To further evaluate the cell compatibility of mTG sponges, ADSCs were cultured and inoculated into the scaffold. Cell proliferation and viability were measured via alamarBlue assay and LIVE/DEAD fluorescence staining, respectively. Cell adhesion on the sponges was observed by scanning electron microscopy (SEM).

## Materials and Methods

### Preparation of gelatin sponges

Gelatin sponge is a freeze-dried product of gelatin hydrogel. Thus, the gel needs to be prepared first. The mTG crosslinked gelatin hydrogel was prepared as described in our previous publication (Yang et al., 2016). Gelatin powder (type A, 300 Bloom; Sigma, St. Louis, MO, USA) was dissolved in deionized water at 50 °C to make 4% solution and sterilized through a 0.22 μm syringe filter. mTG powder (enzymatic activity >100 U per gram; Bomei, China) was dissolved in phosphate-buffered saline (PBS) to make 10% solution and also sterilized through a syringe filter. Gelatin hydrogel was obtained by mixing gelatin and mTG solution with a ratio of 40 μL of mTG per milliliter gelatin solution. The mixture was incubated at 37 °C for gelatinization. The resultant hydrogel was frozen at −20 °C for 8 h and freeze dried for 48 h to produce the mTG sponges. Un-crosslinked gelatin sponge was made by cooling the 4% gelatin solution at 4 °C for 2 h, freezing at −20 °C for 8 h, and freeze drying for 48 h. Samples of the aforementioned sponges in dry and wet states (after immerging in PBS for 2 h) were observed under a stereoscopic microscope (Jinteng, Tianjin, China) attached to a CMOS camera (MD50; Guangzhou Ming-Mei Technology Co., Ltd., Guangzhou, China).

### Porosity of mTG sponges

The porosity of gelatin sponges was measured by a liquid-displacement method described in the literature (Kanokpanont et al., 2012). A pre-weighed mTG sponge was placed into a known volume (*V*_1_) of absolute ethanol and degassed for 5 min by a vacuum pump. The full volume of ethanol and the soaking sponge was recorded as *V*_2_. The ethanol-soaking sponge was then abandoned, and the remaining volume of ethanol was recorded as *V*_3_. The porosity (ε_1_) of the sponge was calculated as follows: 

(1) \begin{eqnarray*}{\varepsilon }_{1}=({V}_{1}-{V}_{3})/({V}_{2}-{V}_{3})\times 100\text{%}\end{eqnarray*}

### Uniaxial tensile test

The mechanical property of dry and wet mTG sponges was measured on a uniaxial mechanical testing apparatus (HPE; Yueqing Handpi Instruments Co., Ltd, Wenzhou, Zhejiang, China) equipped with 20 N capacity. Wet sponges were prepared by dipping the dry sponges in PBS for 1 h. Rectangular samples (20 mm × 5 mm) were prepared from the dry and wet mTG sponges prior to testing, and the sample thickness was measured by a vernier caliper. A small tare load of 0.01 N was applied to ensure that each sample received the same initial tension. Samples were tested up to failure at a crosshead speed of 10 mm/min. Data on stress versus strain were calculated from the measurements. The linear slope of the curve was calculated to obtain the elastic modulus. The ultimate tensile stress and elongation at break were also determined. 

### Water absorption

To measure the water absorption capacity of mTG sponges, a pre-weighed freeze-dried sponge (*W*_0_) was immersed into deionized water at room temperature for 1 h. The water-absorbing sponge was taken out. Excess water on its surface was carefully removed using filter paper, then weighed again. The weight (*W*_1_) was then noted. The water absorption ratio (ε_2_) was calculated as follows: 

(2) \begin{eqnarray*}{\varepsilon }_{2}=({W}_{1}-{W}_{0})/({W}_{0})\times 100\text{%}\end{eqnarray*}

### Material degradation

#### Hydrolysis test

The hydrolysis performance of the mTG sponges was assessed by immersing the materials in deionized water for a certain period. Their degradation rates were also analyzed. Pre-weighed freeze-dried mTG sponges were sterilized in 75% ethanol for 20 min, followed by 30 min UV irradiation and several washings with deionized water. The sterilized sponges were kept in water and placed in a cell incubator at 37 °C. At the specified time points (i.e., one, two, and three months), the remaining sponges were obtained, freeze dried, and weighed again. The percentage of remaining weight divided by original weight is the material hydrolysis rate. **Enzymolysis test:** Pre-weighed wet sponges were exposed to 0.1% collagenase type I (>125 CDU/mg; Invitrogen, Carlsbad, CA, USA) at 37 °C for 6 h in a horizontal shaker. The remaining sponges were obtained and weighed at the specified time points. The extent of enzymatic degradation was determined by calculating the percentage of the remaining weight versus the original weight. Sponges immersed in PBS served as negative control.

### Subcutaneous implantation

Animal study was approved by the Institutional Animal Care and Use Committee (IACUC) of Sichuan University, all experiments were performed in accordance with the guidelines of IACUC of Sichuan University. *In vivo* biocompatibility of the mTG sponges was assessed in subcutaneous implantation of Sprague–Dawley (SD) rats (age 7 −8 weeks) as described in our previous publication (Yang et al., 2016). The mTG sponges were sterilized in 75% ethanol, followed by UV irradiation and several washings with PBS, and equilibrated in DMEM for 12 h. The sponges were then cut into a size of 7 mm × 7 mm × 4 mm using a scalpel in a sterile bio-safety cabinet and surgically placed within subcutaneous pockets located on the dorsum of SD rats. Each of the four rats in the experimental group received two dorsal subcutaneous implants. Meanwhile, two rats in the control group were subjected to surgery, but no sponge was implanted. The rats were sacrificed at the designated time points (one and three months). The implanted sponges were resected from the underlying muscle, and their sizes were measured using a Vernier caliper. For material degradation analysis, the harvested implant was cut to expose its cross section for imaging. Images were analyzed using Image Pro Plus software (Media Cybernetics, Rockville, MD, USA). The average thickness of the coating tissue on the sponges was measured, and the volume of the coating tissue was estimated. The volume of the remaining sponges was calculated by subtracting the volume of the coating tissue from the full volume of the implants. The extent of *in vivo* biomaterial degradation was determined by calculating the percentage of the remaining volume versus the original volume of the sponges.

### Cell proliferation assay

The isolation method of primary ADSCs was described in our previous publications (Li et al., 2015; Yang et al., 2016). mTG sponges were prepared as mentioned in the previous section. All of the sponges (7 mm × 7 mm × 4 mm) were aspirated dry before cell seeding. The sponges were then placed in each well of a 24-well plate. Each gelatin sponge was seeded with 100 μL of ADSC suspension solution (∼5.0 ×10^4^ cells). After 1 h of incubation at 37 °C with 5% CO_2_, the culture medium was supplemented to each well. The culture was conducted for a month and the medium was replaced twice a week. Cell growth was observed daily using an inverted microscope (CKX41; Olympus, Tokyo, Japan). At each time point, the detected samples were refreshed with culture medium supplemented with 10% alamarBlue solution (Yeasen, Shangha, China). After 3 h of incubation, the incubation solutions were transferred to a 96-well plate, and fluorescence was measured with a plate reader using excitation/emission wavelengths of 530/590 nm. ADSCs seeded on culture flask (25 cm^2^) served as negative control.

### LIVE/DEAD cell assay

The survival status of ADSCs in mTG sponges was detected by LIVE/DEAD staining. Cell/sponge constructs were prepared (see section ‘Cell Proliferation Assay’) and cultured for a month. The constructs were washed in PBS and cut at 1-mm intervals along the transverse planes of the sponge. Representative slices from the middle of the sponge were incubated at 37 °C for 30 min in a solution containing 4 μM calcein-AM (Sigma, St. Louis, MO, USA) and 4 μM propidium iodide (Sigma, St. Louis, MO, USA). After incubation, samples were washed and observed at an inverted fluorescent microscope (XDS30; Sunny Optical Technology, Yuyao, Zhejiang, China) equipped with a digital camera.

### SEM observation

To study cell adhesion on scaffold, SEM images were taken from the slices of cell/sponge constructs (see previous section). The samples were washed with PBS and fixed in 2.5% glutaraldehyde for 2 h at room temperature. Scaffolds were then dehydrated with gradient ethanol aqueous solutions (10%–100%) and dried overnight under vacuum. Before observation, all of the specimens were coated with gold and observed under a scanning electron microscope (Hitachi S3400+EDX; Hitachi, Tokyo, Japan) at an accelerating voltage of 20 kV.

### Statistical analysis

Data are presented as mean ± SD. Statistical analyses were performed using SPSS software (version 14.0). Statistical significance was evaluated using one-way ANOVA with least significant difference test. The level of statistical significance was set at *P* < 0.05.

## Results

### Material appearance and physical characteristics

**Figure 1:**
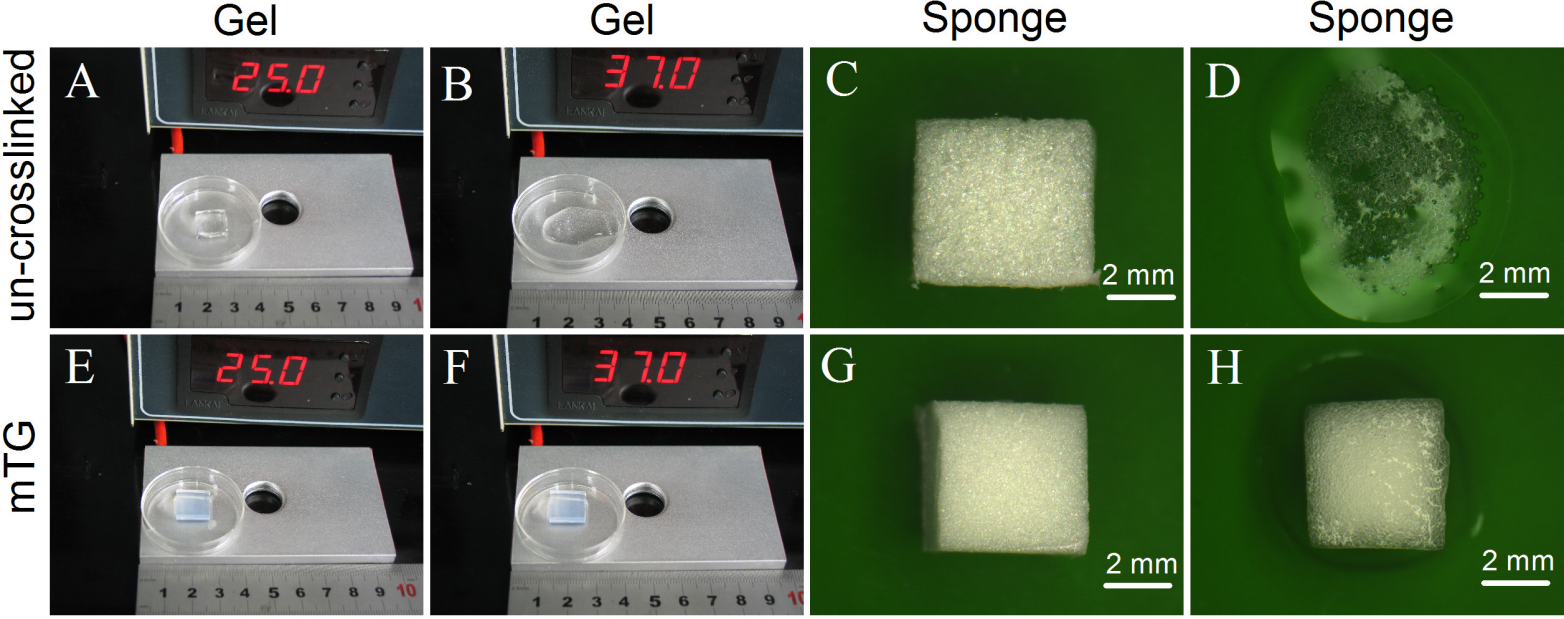
Appearance of gelatin gel and sponge. The morphology of un-crosslinked gelatin gel on a thermostat aluminum stage at 25 °C (A) or at 37 °C (B); the un-crosslinked gelatin sponge at 25 °C (C) or at 37 °C (D); the mTG crosslinked gelatin gel at 25 °C (E) or at 37 °C (F); and the mTG crosslinked gelatin sponge at 25 °C (G) or at 37 °C (H).

The un-crosslinked gelatin hydrogel is colorless. It can maintain a gel state at 25 °C but melt at 37 °C. Figures 1A and 1B show the morphological change of the hydrogel. After crosslinking by mTG, the gelatin hydrogel showed a milky white color. It can maintain a gel state either at 25 °C or at 37 °C (Figs. 1E and 1F, respectively). Both the crosslinked and un-crosslinked hydrogels can be freeze dried into white porous sponges (Figs. 1C and 1G, respectively). After freeze drying, the stiffness of the gelatin material increased significantly. However, the un-crosslinked gelatin sponge is considerably brittle. Once the sponge is exposed to water, its material structure begins to collapse (Fig. 1D). Therefore, it cannot be used as a scaffold material. The mTG sponge can maintain its porous scaffold structure even under 37 °C warm water. However, it is gradually restored to the porous hydrogel after water absorption (Fig. 1H).

One of the major advantages of sponge scaffold is its high porosity. The high porosity contributes to cell growth and promotes the exchange of nutrients and metabolites. The porosity of mTG sponge material was 53.51 ± 3.45%, and the pore size of the sponge is small and even. Moreover, as gelatin sponge has high hydrophilicity, it can absorb water more than its own weight. Thus, it is often used as a hemostatic sponge. The water absorption rate of the mTG sponge is 1,209.3 ± 57.8%. Notably, although the weight of the sponge increased by more than 10 times after water absorption, the sponge decreased in volume. On the one hand, the surface tension of water molecules in the sponge is strong, leading to a certain degree of shrinkage. On the other hand, the material has good hydrophilicity.

**Figure 2:**
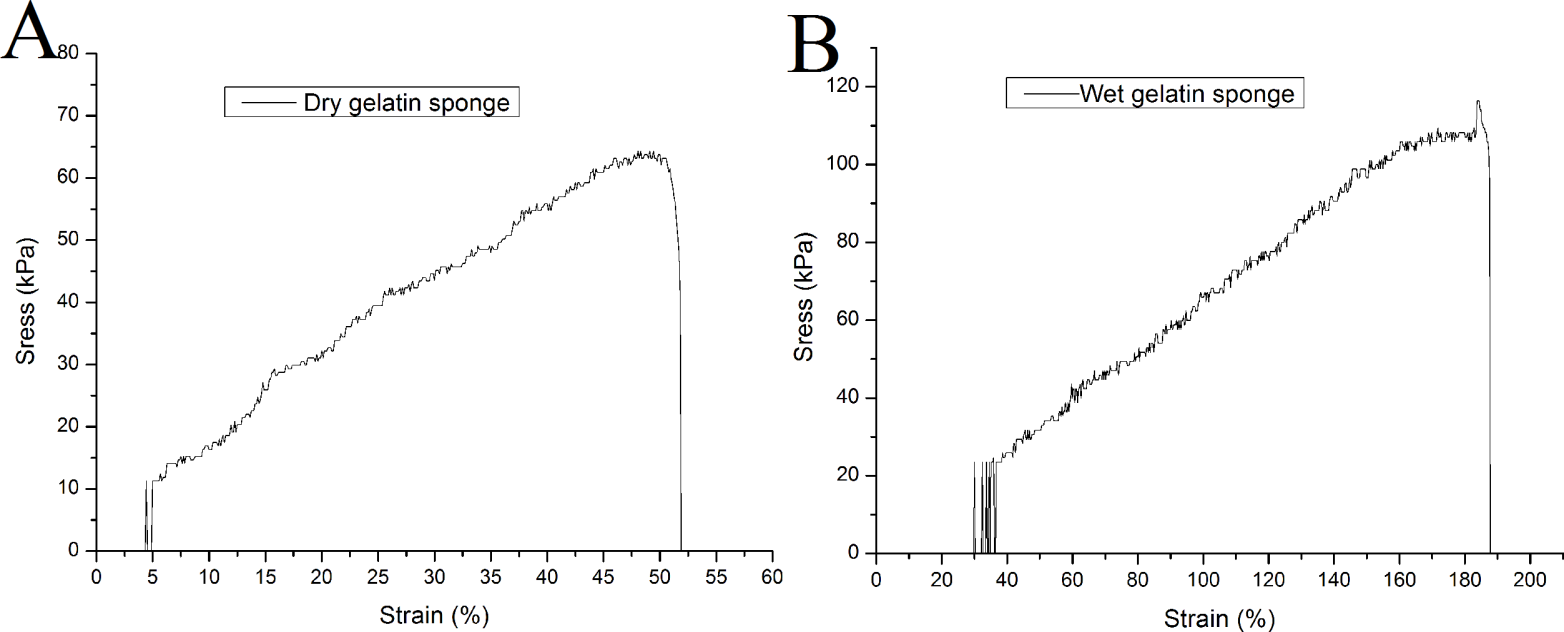
Uniaxial mechanical testing of mTG sponge. (A) Tensile curve of dry gelatin sponge and (B) tensile curve of wet gelatin sponge.

The elastic modulus is an important index for measuring the strength of a material. In the uniaxial tensile test, the gelatin sponge scaffold has a relatively high elastic modulus in dry state, which is 133.4 ± 7.9 kPa. It can bear a maximum tensile force of 67.9 ± 8.1 kPa, and its elongation at break is 57.1 ± 10.0%. When the material absorbs water, its elastic modulus decreases to 59.9 ± 3.0 kPa (*P* < 0.01, when compared to that of the dry sponges). The maximum compressive capacity was 110.8 ± 5.4 kPa (*P* < 0.01), and the elongation at break was 177.2 ± 6.2% (*P* < 0.01). Representative tensile curves of dry and wet gelatin sponges were shown in Fig. 2.

### Hydrolysis and enzymolysis

**Figure 3:**
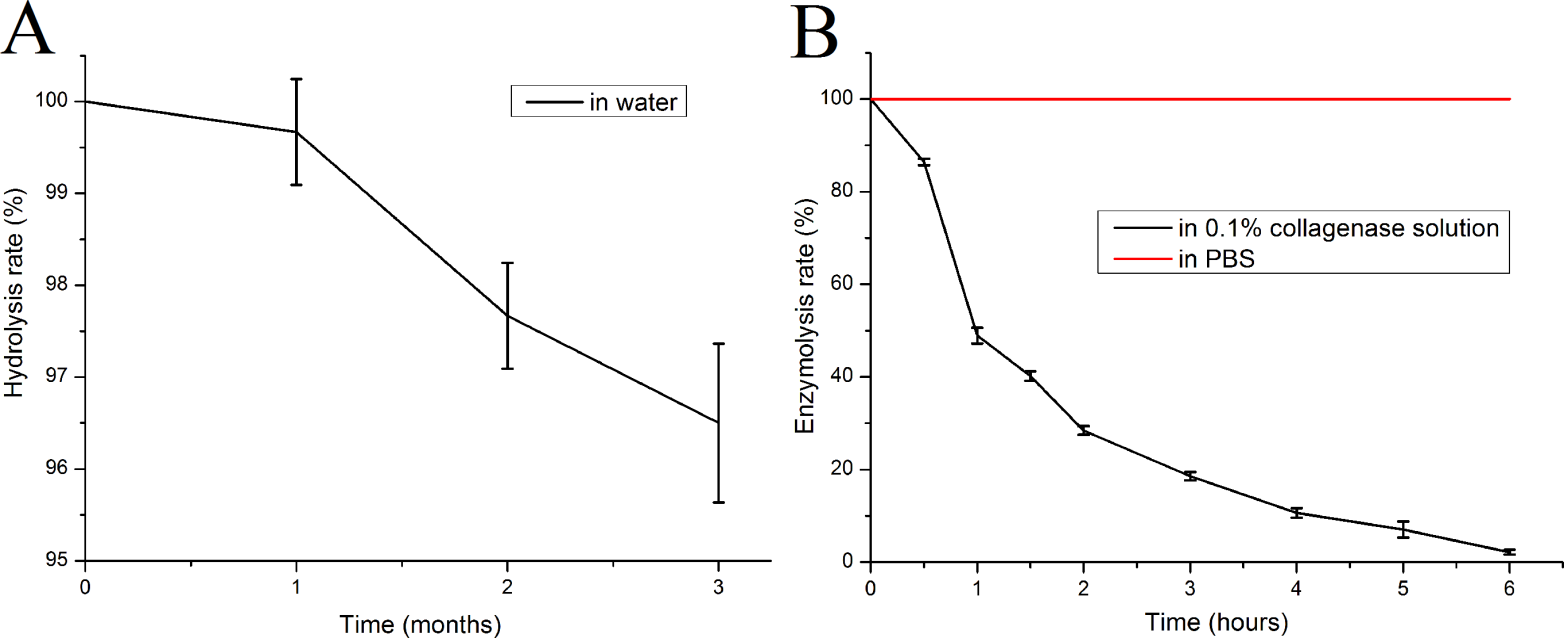
*In vitro* degradation performance of mTG sponges. (A) Hydrolysis curve and (B) enzymolysis curve.

The mTG sponge is highly resistant to hydrolysis. After three months of soaking in water at 37 °C, its mass loss became less than 5% (Fig. 3A). However, the sponge is sensitive to collagenase. Under the catalysis of 0.1% collagenase type I, the quality of the mTG sponge is decreased exponentially, and it can be completely dissolved after only about 6 h, whereas no obvious mass loss could be seen in control group (Fig. 3B). Gelatin is a product of collagen hydrolysis, and collagenase is a digestive enzyme that is specific for collagen and collagen derivatives. Therefore, gelatin sponges can be degraded by collagenase solution.

### Analysis of subcutaneous implantation

**Figure 4:**
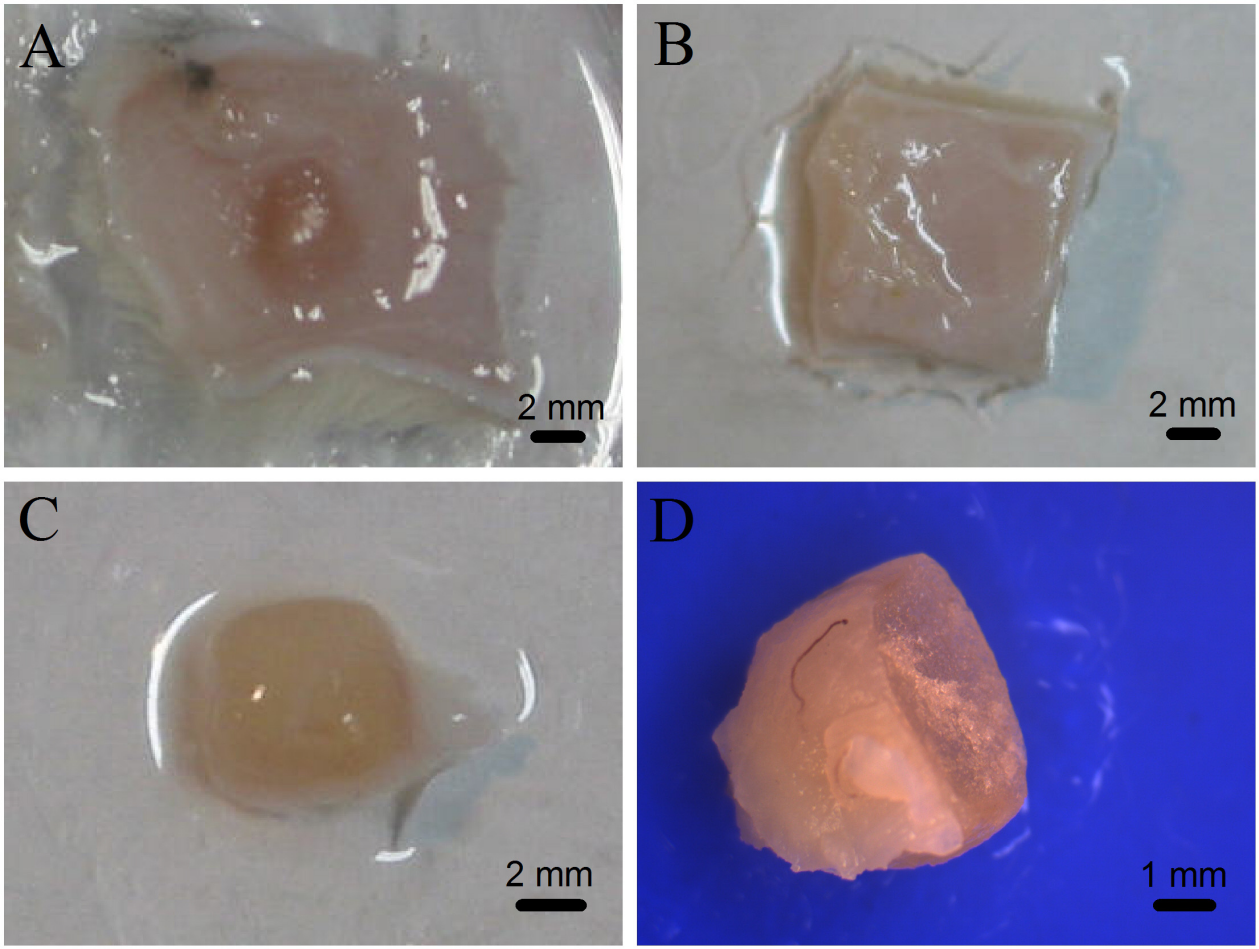
Photographs of gelatin sponges implanted subcutaneously in rats. The implanted gelatin sponges with attached skin were removed from the experimental rats after one month of subcutaneous implantation (A) or after three months of subcutaneous implantation (B); the one-month-old implant without attached skin (C); and cross section of the one-month-old implant was observed under a stereomicroscope (D).

The mTG sponges were implanted subcutaneously in rats to evaluate the degradation of the material *in vivo* and observe whether they would cause inflammatory responses. The results showed that all animals in the experimental and control groups had no inflammatory reaction after operation. Figure 4A shows the morphology of the material in the subcutaneous tissue after one month of implantation. The implant is located between the skin and the muscle layer, the surface of which is wrapped by a connective tissue. No inflammatory reaction nor pus was observed. After three months of implantation, the mTG sponges were completely degraded and absorbed without causing an inflammatory response, as shown in Fig. 4B.

To assess the tissue rejection in the subcutaneous implantation, the one-month-old implant was removed and analyzed, as shown in Fig. 4C. The implant is like a flesh-color ellipsoid. The implant material is cuboid, and then becomes ellipsoid, indicating that after implantation, the material has shown a certain degree of degradation at least on its edge. Subsequently, the implant was centrally cut to observe and measure the thickness of the coating layer and calculate the remaining volume of the sponge. After one month of implantation, the average residual volume of mTG sponges was 27.73 ± 2.68%, and the average thickness of their coating tissue was 0.19 ± 0.16 mm. In addition, tiny capillaries could be seen on the surface of some implants under observation of a stereomicroscope (Fig. 4D). Accordingly, the material can promote angiogenesis.

**Figure 5:**
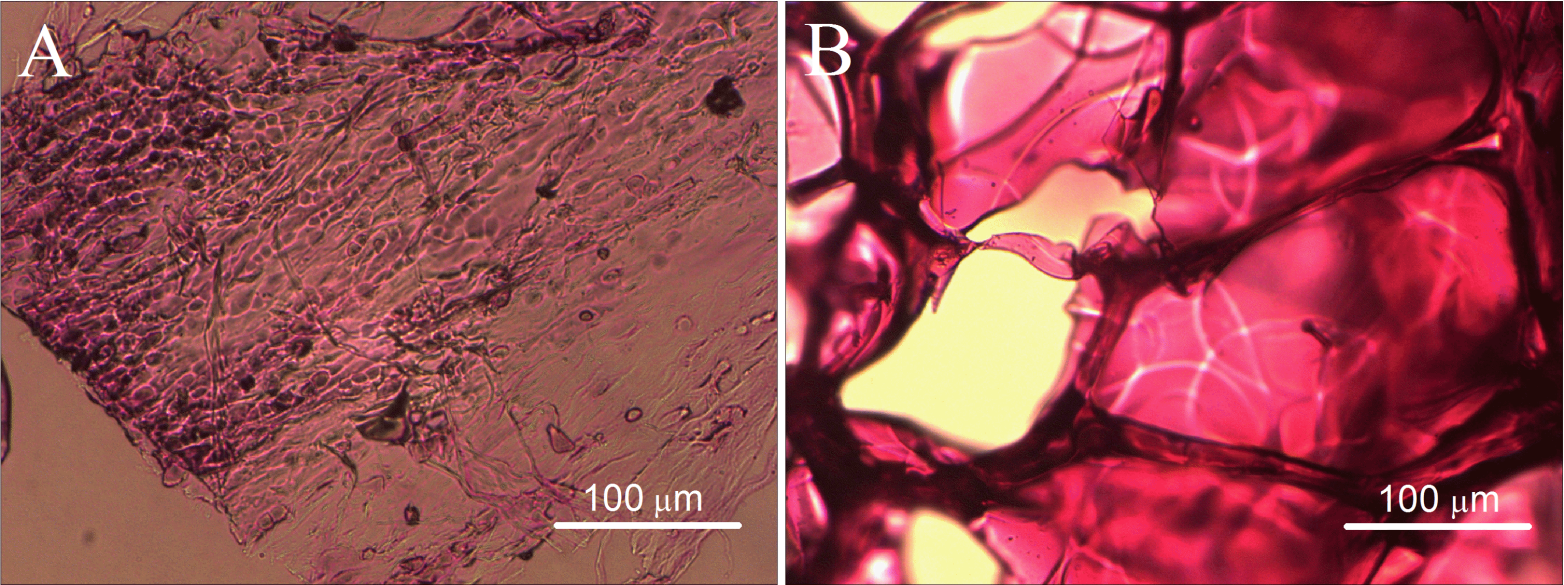
Histological observations of the gelatin sponges after one month of subcutaneous implantation in rats. (A) H&E staining of marginal slice of the implant and (B) H&E staining of the central slice of the implant.

In the images of H&E staining, we observed an abundance of cells and fibrous tissue in the coating tissue of the implant, including a few of the megakaryocytes, as shown in Fig. 5A. In the central slices of the implant, only the gelatin sponge material can be seen, whereas no cells were observed (Fig. 5B). This condition can be explained by the fact that the implantation time is short; the cells have not yet penetrated into the interior of the material.

### Observation cell growth via inverted optical microscope

**Figure 6:**
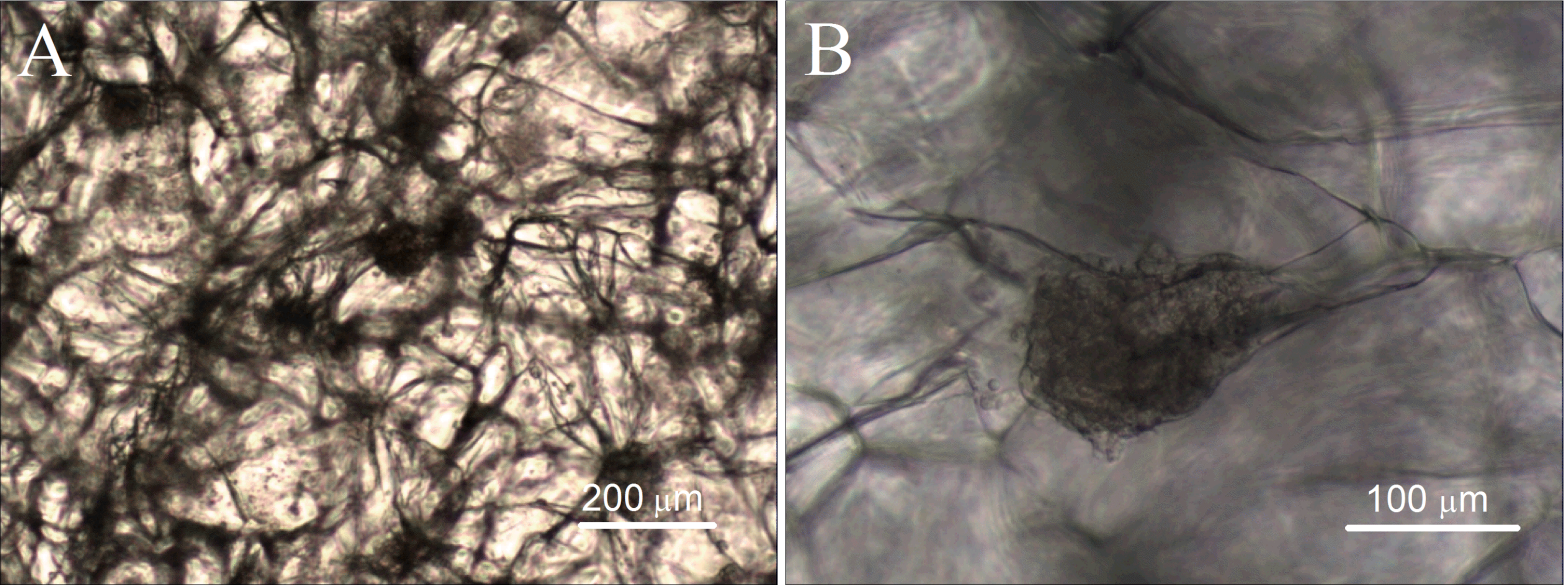
Cell growth in mTG sponges observed under optical microscope. (A) The cell morphology and distribution were observed under a microscope field of 4×, and (B) the adhesion of the cells on the scaffold was observed under a microscope field of 10×.

The cell compatibility of gelatin sponge was detected by cell/scaffold co-culture. The ADSCs were seeded into the scaffold. After one month of culture, the cell growth and morphology were observed using an inverted optical microscope. Observation via microscope clearly shows that the gelatin sponge contains numerous fiber connections, which form the interconnected holes and mesh structures. The interconnected meshes facilitate cell growth and the delivery of nutrients, and many cells are evenly distributed at all levels of the material, as shown in Fig. 6. The cells are tightly attached to the gelatin fibers and grow up into many cell aggregates, which vary in size and shape. In addition, some of the cells grow laterally, covering many meshes and forming thin cell sheets.

### Evaluation of cell proliferation

**Figure 7:**
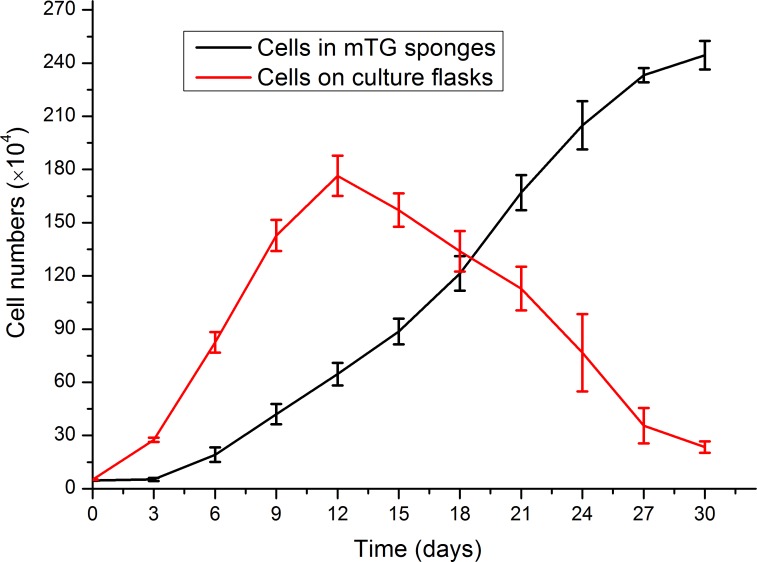
Evaluation of cell proliferation in mTG sponges and on culture flasks.

AlamarBlue assay was used to investigate the effect of gelatin sponge on cell proliferation. From 2D to 3D culture, a period for cell adaptation was allowed to cope with the environmental changes. During this period, the cellular behaviors were mainly adhesion and migration, whereas the cell proliferation was not evident. The number of cells in mTG sponges began to increase on the third day after inoculation. On Day 30, the number of cells was approximately 50 times that of the original number. Therefore, the material can support cell adhesion and proliferation. The cell growth curve is shown in Fig. 7. The mTG sponge has many spaces for cell accommodation. Thus, we speculate that with the extension of culture time, cell growth will continue. In control groups, the cells apparently showed a higher proliferation rate compared with cells cultured in sponges from day 0 to day 12. However, the cell number on culture flasks almost linearly decreased after 12 days of culture, as the cells stopped growth caused by cell contact inhibition, and some cells were detached from the flask surface.

### Analysis of LIVE/DEAD cell fluorescence staining

**Figure 8:**
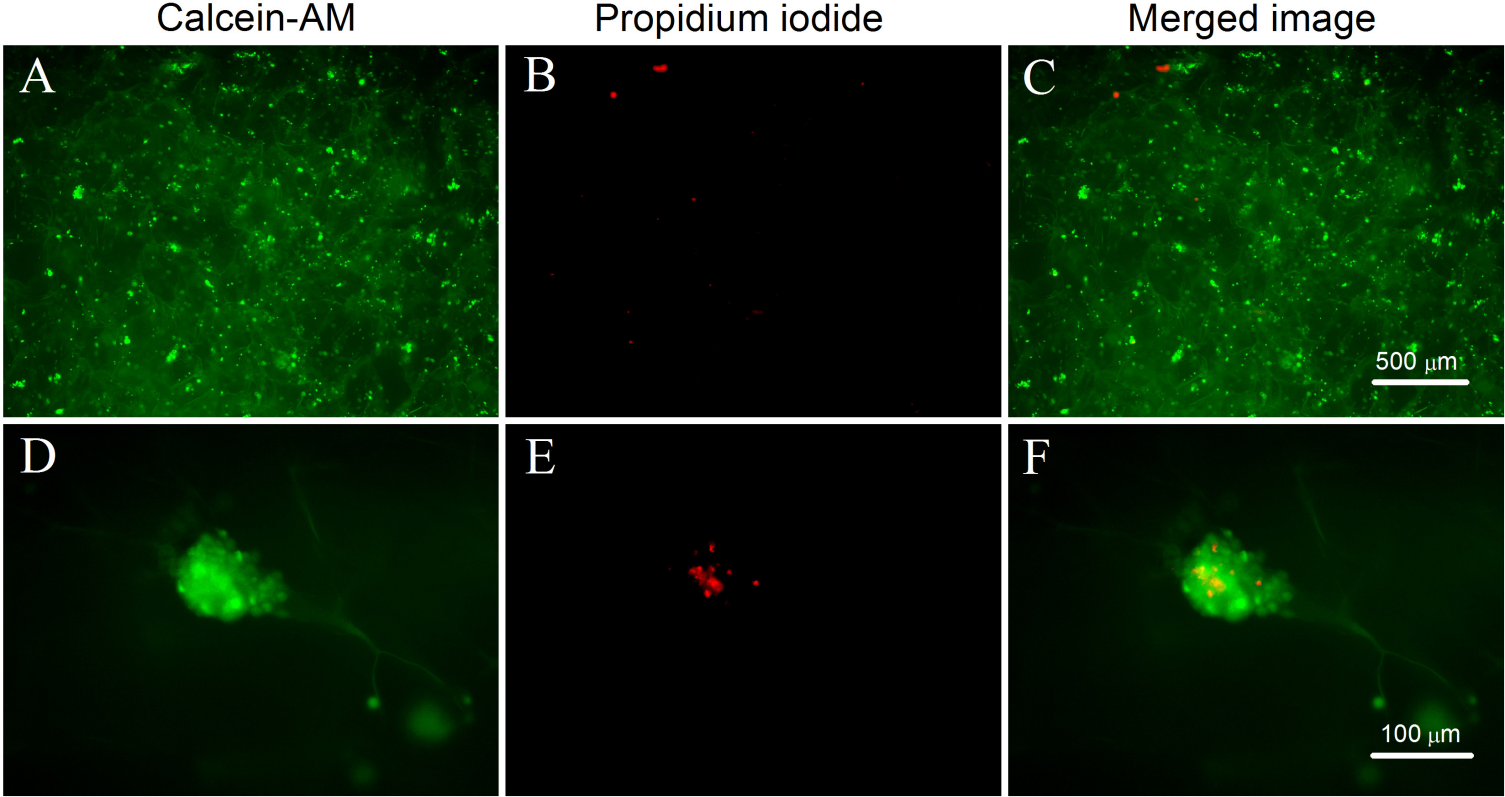
LIVE/DEAD cell fluorescence staining on mTG sponges. (A and D) Live cells are stained with calcein-AM (green), (B and E) dead cells are stained with propidium iodide (red), and (C and F) merged fluorescence images of cellular LIVE/DEAD staining. Magnification of the microscope is (A–C) 4× and (D–F) 20×.

The survival and distribution of the cells on the mTG sponge can be observed directly by LIVE/DEAD cell fluorescence staining. Figures 8A–8C show that the cells are evenly distributed in the sponge scaffold. After one month of culture, the cells formed numerous cell aggregates in the material, which is consistent with the observation under the light microscope. Figure 8A shows that living cells are numerous and distributed throughout the material. The number of apoptotic cells is small. Only a few scattered red dots can be seen in the picture (Fig. 8B). Next, a high-powered microscope was used to observe the viability of the cell aggregates. We found that some of the cell aggregates showed strong green fluorescence, indicating an accumulation of many living cells (Fig. 8D). Within the aggregate, a small number of cells were dead (Fig. 8E). This condition may be due to the high cellular density in the center of the aggregate, which hinders the metabolism and leads to cell apoptosis. The results of LIVE/DEAD staining further confirmed that the mTG sponge had good cell compatibility.

### Observation cell adhesion by SEM

**Figure 9:**
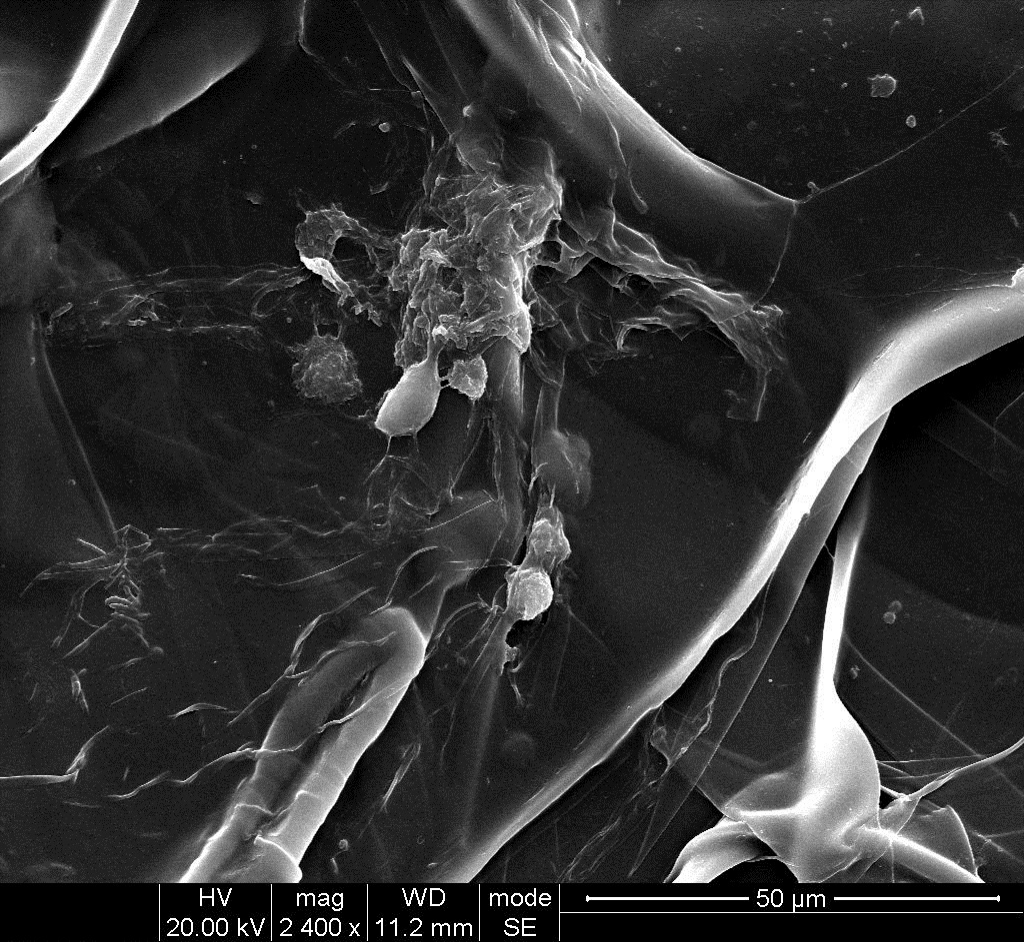
SEM observation of cell adhesion on mTG sponge.

The cell adhesion and morphology on the mTG sponge was observed by SEM. Figure 9 shows that the scaffold material presents a lamellar structure that forms large pores. The approximative pore size based on the SEM images of mTG sponge is 100 μm. We estimate that each large pore can accommodate dozens of cells. Some of the cells on the material surface grow together to form a cell aggregate. Other cells are tightly attached to the surface of the material. In addition, several cells show a small ball shape. Thus, we speculate that these are the cells undergoing cell division.

## Discussion

mTG is a promising material crosslinking agent. First, the enzyme-mediated crosslinking reaction can be achieved under physiological conditions, which is particularly suitable for the formation of *in situ* injectable hydrogels. Second, the residual enzyme in the crosslinking reaction will be degraded *in vivo*, and the harm to the cell is substantially less than that of the chemical crosslinking agent. Actually, mammalian transglutaminases (TG2) are found extensively in the extracellular matrix of animal tissues; they act as a matrix stabiliser through protein crosslinking activity and as an important cell adhesion protein involved in cell survival (Collighan & Griffin, 2009). As a homologous enzyme of TG2, mTG has good biocompatibility. Small amounts of residual mTG in hydrogels exhibit no cytotoxicity (Schloegl et al., 2012). Third, mTG can selectively recognize specific amino acid sequences of gelatin materials and form a stable interconnection between protein molecules. Consequently, the physical and chemical properties of the crosslinked materials were improved.

In the preparation and application of gelatin sponge scaffolds, the stiffness and strength of materials, porosity and permeability, hydrophilicity, biocompatibility, as well as biodegradability and vascularization should be considered.

Stiffness and strength are important parameters of biomaterials. The stiffness of the material significantly influences cell growth and differentiation (Xia, Liu & Yang, 2017). The appropriate material stiffness can provide good mechanical support for cells and tissues. However, the high stiffness will make the material brittle and easily breakable. The ideal biomaterial is designed according to the stiffness of the replaced tissue. For example, scaffold materials for bone tissue engineering should have a high stiffness, and the stiffness of the materials used for skin repair should be relatively small. In the dry state, the gelatin sponge scaffold has high stiffness. However, when the material is immersed in aqueous solution, its stiffness decreases significantly. Therefore, the pure gelatin sponge scaffold is more suitable for soft tissue repair. In this case, the main concern for the material is its strength. The strength of sponge scaffolds can be characterized by measuring the elastic modulus and yield stress. Moreover, the strength of the material can be adjusted by changing the manufacturing process, crosslinking method, and polymer concentration. Hence, the strength of gelatin sponge can be adjusted by changing the concentration of gelatin solution or the amount of crosslinking agent.

The pore size and porosity of materials affect the cellular migration, growth, and proliferation (Wang et al., 2016). In sponge scaffolds, the interconnected porous structure contributes to the transport of nutrients and metabolites, leading to faster cell growth and proliferation. In particular, the large pore size is beneficial to the ingrowth of cells on the material. The pore size and porosity of sponge are related to the physicochemical properties of material composition and the manufacturing process. Freeze drying is the most common method for preparing gelatin sponge. The pore size and porosity of gelatin sponge can be adjusted by changing the gel concentration, freezing temperature, and freezing time. The gelatin sponge usually has high porosity, which makes it suitable for use as a tissue engineering scaffold. However, the pore size of the sponge should be determined according to the actual requirement. At present, the optimal pore size is yet to be determined. Some studies have suggested that scaffold material with a small pore size is optimal because it is closer to the nanostructure of natural tissue. Other studies suggested that the large pore structure may be more beneficial for cell proliferation and ECM secretion (Griffon et al., 2006; Nava et al., 2016). In addition, studies have shown that cells in material with small pore size will undergo dedifferentiation, whereas those in the large pore size stop proliferating and secrete more ECM instead (Lien, Ko & Huang, 2009).

The gelatin sponge has high hydrophilicity. It can absorb water weighing more than 10 times of its own. Thus, the gelatin sponge is often used as hemostatic material in clinics. Although the highly hydrophilic scaffold material is beneficial for cell growth and proliferation, the high water absorption causes the scaffold to expand excessively and accelerates its degradation. As the high concentration of gelatin can form more compact hydrogel, changing the gelatin concentration can adjust the water absorption rate of the mTG sponge to stabilize its mechanical structure.

Biocompatibility refers to the ability of scaffolds to support cell growth and proliferation without causing any toxic or immune response. It is also defined as the interaction between biomaterials and adjacent natural tissues. The ideal scaffold material does not cause rejection after implantation *in vivo*. In the current study, we implanted mTG sponges into the subcutaneous layer of rats. One month after implantation, no inflammation was observed at the implant site. However, the mTG sponge was wrapped by a thin layer of connective tissue, indicating that the material has some minor tissue rejection in the early stage of implantation. Along with the prolongation of implantation time, the connective tissues and the implanted materials will be absorbed by the host. Consequently, the coating layer was not observed, and the materials were absorbed after three months of implantation.

In the field of regenerative medicine, the scaffold material usually needs to be biodegradable. Material degradation occurs in some biological processes, such as enzymolysis or hydrolysis, resulting in the destruction of the material structure or the loss of material functional groups. If the material degradation rate is properly designed, the newborn tissue will fill into the room left by the degraded material. This condition will not cause the collapse of the material structure. Therefore, the degradation rate of an ideal material needs to be equal to or comparable to the regeneration rate of the newborn tissue. However, some exceptions exist, such as regenerative bone tissue, articular cartilage, or cornea, which do not require complete degradation of the implanted material. For these applications, the most important requirement is that the implant material should be firmly combined with the surrounding tissue (Slaughter et al., 2009). For biodegradable materials, another point must be considered, that is, the final condition of the material degradation products. Ideally, the degradation products need to be absorbed by the host or excreted by metabolism. If this case is not possible, the degradation products should at least be non-cytotoxic or not trigger the immune response.

Angiogenesis is an important criterion for evaluating the performance of scaffold materials. Almost all of the tissues in the body, except the cartilage and cornea, require blood supply for growth and development. Therefore, biomaterials designed by mimicking the natural tissue should have the ability to promote angiogenesis. For the porous scaffolds implanted in the body, the degree of vascularization is related to the pore size, porosity, degradation rate of the material, as well as the secretion factors of the host (Walthers et al., 2014). To improve the degree of vascularization of scaffold materials, growth factors, such as vascular endothelial growth factor, fibroblast growth factor, or drugs, should be added to promote angiogenesis (Martino et al., 2015). When the material is degraded, the factors or drugs embedded in the material will be released. In the mTG sponges of subcutaneous implantation, some capillaries form on the coating tissue. Results show that these sponges are beneficial to angiogenesis. In addition, ADSCs have the potential to differentiate into adipocytes, osteoblasts, chondrocytes, cardiomyocytes, vascular cells, and endothelial cells. The combination of gelatin sponge scaffolds with ADSCs may further promote the vascularization of sponge scaffolds by cell autocrine or paracrine effects. Moreover, we speculate that inflammatory response following the biomaterial implantation may also contribute to the angiogenesis. Certainly, more studies are needed to uncover the underlying mechanism of angiogenesis. Taken together, we believe that gelatin sponges encapsulated with cells or drugs will have great potential for tissue regeneration.

##  Supplemental Information

10.7717/peerj.3665/supp-1 Data S1 Raw data Experimental data

## Additional Information and Declarations

### Data Availability

The following information was supplied regarding data availability: The raw data is provided as Data S1.
